# The long-term effect of removing the UV-protectant usnic acid from the thalli of the lichen *Cladonia foliacea*

**DOI:** 10.1007/s11557-022-01831-y

**Published:** 2022-09-01

**Authors:** Katalin Veres, Mónika Sinigla, Krisztina Szabó, Nóra Varga, Edit Farkas

**Affiliations:** 1 Institute of Ecology and Botany, Centre for Ecological Research, Alkotmány u. 2–4, Vácrátót, H-2163 Hungary; 2 Bakony Museum of the Hungarian Natural History Museum, Rákóczi tér 3–5, Zirc, H-8420 Hungary

**Keywords:** Lichen-forming fungi, Symbiosis, Photoprotection, Acetone rinsing, Seasonality, Lichenicolous fungi

## Abstract

Terricolous lichens are abundant in semi-arid areas, where they are exposed to high irradiation. Photoprotection is essential for the algae as the photobiont provides the primer carbon source for both symbionts. The UV-protectant lichen metabolites and different quenching procedures of the alga ensure adequate photoprotection. Since the long-term effect of diminishing UV-protectant lichen metabolites is unknown, a major part of lichen secondary metabolites was removed from *Cladonia foliacea* thalli by acetone rinsing, and the lichens were then maintained under field conditions to investigate the effect on both symbionts for 3 years. Our aim was to determine if the decreased level of UV-protectant metabolites caused an elevated photoprotection in the algae and to reveal the dynamics of production of the metabolites. Photosynthetic activity and light protection were checked by chlorophyll *a* fluorescence kinetics measurements every 6 months. The concentrations of fumarprotocetraric and usnic acids were monitored by chromatographic methods. Our results proved that seasonality had a more pronounced effect than that of acetone treatment on the function of lichens over a long-term scale. Even after 3 years, the acetone-treated thalli contained half as much usnic acid as the control thalli, and the level of photoprotection remained unchanged in the algae. However, the amount of available humidity was a more critical limiting environmental factor than the amount of incoming irradiation affecting usnic acid production. The lichenicolous fungus *Didymocyrtis cladoniicola* became relatively more abundant in the acetone-treated samples than in the control samples, indicating a slight change caused by the treatment.

**Supplementary Information:**

The online version contains supplementary material available at 10.1007/s11557-022-01831-y.

## Introduction

Terricolous lichens are characteristic and important members of biological soil crusts, dominating arid and semi-arid ecosystems (Belnap and Lange 2003). These habitats exhibit intense irradiation and UV radiation, temperature extremes, low relative humidity, and precipitation (Borhidi et al. 2012; Dövényi 2010). Terricolous lichens have to cope with these harsh environmental conditions. Furthermore, according to various climate change scenarios, the climate may change in the direction of further extremities. The climate will be drier and warmer in summer and more humid in winter as in the case of Hungary for example (Kocsis et al. 2018). Lichens are extremotolerant organisms, several of which are cosmopolitan and exist under a wide range of environmental conditions; therefore, they are expected to tolerate environmental changes forecasted by the predicted scenarios.

Lichens are a stable symbiosis of at least one mycobiont, a photobiont, and an undetermined number of further micro-organisms (Hawksworth & Grube, 2020), resulting in a ‘self-sustaining miniature ecosystem’ (Farrar 1976) by their interactions. The fungi are responsible for the water holding capacity and provide physical (by structure) and chemical (by lichen secondary metabolites) protection against the external influence of the environment, such as herbivory and UV radiation (Molnár and Farkas 2010). Since the photobiont (green algae or cyanobacteria) provides the primary carbon source for both symbionts, the protection of the photosynthetic system is vitally important (Sadowsky and Ott 2016).

High irradiation is one of the most threatening environmental factors, especially when a lichen thallus is wet, since irreversible damage to the photosystem (PS) may occur (Heber et al. 2000). The excitation energy absorbed by the antenna system may be used for photochemical charge separation (photochemical quenching) in the reaction centres until the electron transport chain is saturated. In a saturated electron transport chain, the excitation energy must be removed and can be dissipated as heat or re-emitted as fluorescence (non-photochemical quenching). The non-photochemical quenching via zeaxanthin (Demmig-Adams and Adams 1992; Färber et al. 1997) and desiccation-induced fluorescence quenching (Heber et al. 2001, 2006; Kopecky et al. 2005) are also exhibited in lichens. In the absence of effective dissipation of excessive light energy, the production of by-products, such as damaging reactive oxygen species, can cause irreversible damage to the PSII (Krieger-Liszkay 2005; Müller et al. 2001). In air dry thalli, PSI is not or only partially inhibited by desiccation; therefore, the protection of PSII is critical since photodamage could also occur (Gauslaa and Solhaug 1999; Heber et al. 2010; Solhaug et al. 2003).

Various mechanisms of the photobiont and mycobiont are described for the protection of the lichen thallus against solar radiation damage (Beckett et al. 2021; Gasulla et al. 2012; Kranner et al. 2005; Nguyen et al. 2013; Sadowsky and Ott 2016). The mycobiont is responsible for the reversible drying out for a short period of time. Dehydration protects poikilohydric organisms from excess light (Veerman et al. 2007). The increased accumulation of solar/UV radiation screening pigments (e.g. BeGora and Fahselt 2001; Singh et al. 2011; Solhaug and Gauslaa 1996; Solhaug et al. 2010) and curling during desiccation (Barták et al. 2006) are also effective defence strategies of the mycobiont under high light exposure. The photobiont can protect its photosynthetic apparatus by aggregation of cells and the change in shape during desiccation (De Los Rios et al., 1999; Scheidegger et al. 1995). The increased dissipation of excess light energy by non-photochemical quenching (ΔpH- and zeaxanthin-dependent and desiccation-induced) or conformational change in the chlorophyll-protein complex can also protect the photosystem in the green algal photobiont (Heber et al. 2001; Heber et al. 2007; Paoli et al. 2010; Vráblíková et al. 2006). In the lichen thallus, the symbiotic partners can regulate the photoprotective system of the other symbiotic component (Kranner et al. 2005; Solhaug and Gauslaa 2004).

Acetone rinsing is a method to extract lichen secondary metabolites (LSMs) without damaging the algae in the dry thallus. Solhaug and Gauslaa (1996, 2001) pointed out that both the mycobiont and photobiont were able to survive acetone soaking treatment. Extraction of the lichen substances from dry thalli by acetone was the least detrimental method compared to other solvents (Solhaug and Gauslaa 2001). The treatment did not influence the pigment composition or lipid peroxidation (Candotto Carniel et al. 2017). The results of preliminary experiments (Farkas et al. 2020) showed that *Cladonia foliacea* (Huds.) Willd. survived the longest (1024 h long) acetone treatment, and the long-term tolerance of this species is higher than any of the other 12 species collected and investigated earlier from more humid habitats (Solhaug and Gauslaa 2001). For example, moistened *Xanthoria parietina* re-synthesised part of their parietin content within 3 weeks after acetone rinsing under field conditions (Solhaug et al. 2003). Although several details have been studied in laboratories or short-term experiments, there was no information on the long-term effect of acetone treatment on samples maintained under field conditions for years. The long-term influence of diminishing UV-protectant lichen secondary metabolites from lichen thalli is also unknown. The role of UV-protectant substances in terricolous lichens is more intense because of the high amount of incoming irradiation prevailing over sparse vegetation. Therefore, it was decided to study the long-term effect of usnic acid extraction on the terricolous lichen *Cladonia foliacea*, a species that exhibits a broad ecological tolerance and is abundant in open, dry, sun-exposed habitats in the Hungarian lowland steppe and low mountain rocky grasslands (Verseghy 1974, 1975, 1994). The species can spread easily by rolling due to the force of the wind (Smith et al. 2009) and has a broad thallus water content range optimal for photosynthesis (Mázsa et al. 2002). The species has great potential in experimental applications (Farkas et al. 2020).

The aim of the present investigation was to answer the following questions. Does the decreased amount of UV-protectant metabolites cause an elevated photoprotection in the algae? What are the dynamics of the production of lichen secondary metabolites after acetone rinsing? Will their levels return to their original level under field conditions during several years of experimentation, and how fast do the photobiont and mycobiont of the transplanted samples from a different habitat acclimate in their photoprotection to the changing environment? It was hypothesised that the usnic acid extraction would not cause any serious damage to the algal cells and their photosystem on a long-term scale. It was assumed that because of the lowered level of usnic acid, the algae would take over the photoprotection role of the fungi and increase the level of non-photochemical quenching mechanisms to avoid photodamage. It was expected that the concentration of usnic acid in the treated and transplanted thalli would return to its original control level and local samples within the experimental period and supposedly within 18–20 months (estimation based on Solhaug et al. 2003 and Veres et al. 2022).

## Materials and methods

### Site

Lichen thalli were collected from lowland and mountain sites at the end of summer 2017 in open perennial grassland (*Festucetum vaginatae* Rapaics ex Soó 1929 em. Borhidi 1996) and open dolomite rocky grassland (*Seselio leucospermi-Festucetum pallentis* Zólyomi (1936) 1958) (Borhidi et al. 2012). The two collection sites are in the lowland area of Vácrátót (47.702422 °N, 19.223910 °E) and montane dolomite grassland in the Bakony Mts (Sóly 47.141324 °N, 18.051639 °E), Hungary. Both sites are characterised by a continental climate (mean annual temperature Vácrátót: 11.7 °C, Sóly: 11.17 °C; mean annual precipitation Vácrátót: 442 mm, Sóly: 532 mm) (Hungarian Meteorological Services 2022).

### The research object

**Fig. 1:**
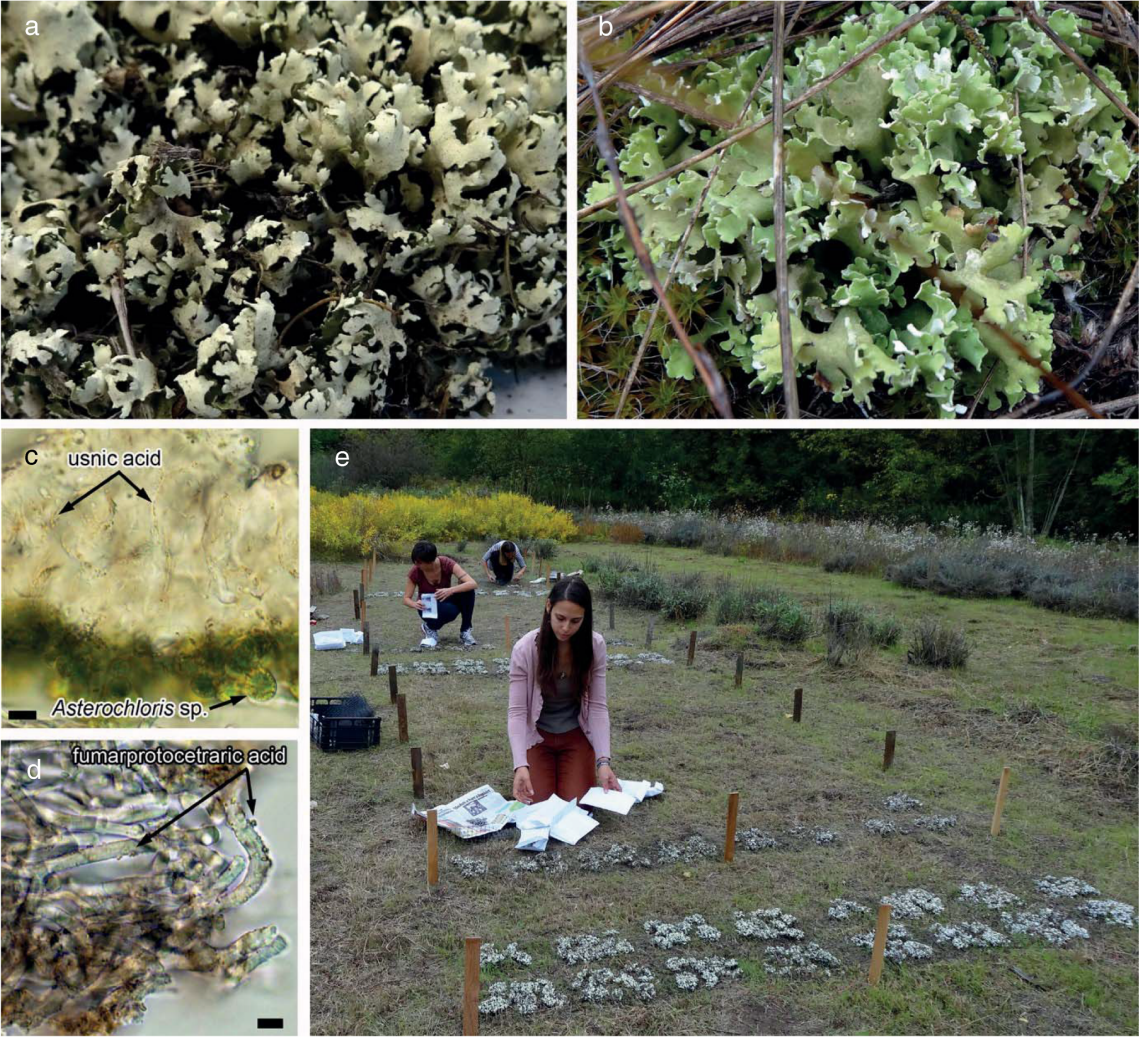
**a**–**e** Dry (**a**) and rehydrated (**b**) thalli of the study object *Cladonia foliacea*; its photobiont *Asterochloris sp*. in the photosynthetic layer (**c**); crystals of usnic acid in the cortex (**c**) and fumarprotocetraric acid in the medulla (**d**) are indicated by arrows. Transplantation (**e**) to the experimental field in National Botanical Garden (Vácrátót, Hungary). Scale bars: **c**–**d** = 10 μm

*Cladonia foliacea* (Fig. 1a, b) is a relatively abundant, terricolous lichen species in Hungary (Verseghy 1974, 1975, 1994) in open, dry, and sun-exposed habitats in lowland steppe and mountain grassland communities. It is widely distributed over Europe and also found in the northern hemisphere temperate region of North America (Smith et al. 2009; Wirth et al. 2013).

The thallus exhibits a thickened upper cortex (Osyczka and Rola 2013) containing usnic acid (Fig. 1c) as a UV-protectant compound. Fumarprotocetraric acid (Fig. 1d) is also detected in the medullary and photosynthetic layer (Farkas et al. 2020b; Hillmann and Grummann 1957; Honegger 1986, 1997, 2012). No lower cortical layer is developed and the photobiont is *Asterochloris sp.*, *Trebouxiaceae* (cf. Škaloud and Peksa 2008).

Usnic acid plays a significant role in protecting the species against the harmful effect of intense radiation and also has other effects such as antibiotics or insecticides (e.g. Cocchietto et al. 2002; Muhoro and Farkas 2021; Nimis and Skert 2006; Seaward 1988; Yilmaz et al. 2004). Usnic acid has a significant role when the species is rehydrated and hence unfolded (Fig. 1b) since the thalli have only a dense, upper cortex; otherwise, the thick, whitish medulla covers them, reflecting the strong solar irradiation (Verseghy 1971). Usnic acid absorbs light and functions as a very effective solar and UV radiation screening pigment (McEvoy et al. 2007; Rancan et al. 2002). The morphology and anatomy were studied by using a Nikon Eclipse/NiU compound microscope (Nikon Corporation, Tokyo, Japan) and a Nikon SMZ18 stereo microscope (Nikon Corporation, Tokyo, Japan). Micrographs were prepared using a Nikon Fi1c camera with NIS-Elements BR ML software (Nikon Corporation, Tokyo, Japan). Voucher specimens were deposited in Lichen Herbarium VBI, Hungary.

### Treatment, field experiment, and collection

After the first collection, the samples were cleaned from plant and moss particles and randomised within each locality (lowland and mountain samples). Then, acetone rinsing was carried out two times for half an hour (Farkas et al. 2020b). After the treatment, the acetone soaked and control samples were placed in the experimental area in National Botanical Garden (Vácrátót, Hungary, 47.705821 °N, 19.229580 °E) for a 3-year experiment (Fig. 1e). An experimental area was chosen where the microenvironmental conditions were quite similar. The thalli were arranged into rows, and every row represented a different treatment. There were no differences between the rows in the shade conditions, vegetation, micro-terrain, or micro-topography. Since the experimental area is situated at a 0.5 km (air distance) from the collection site of the lowland samples, it is considered that these samples are practically placed back in their natural habitat under the same environmental conditions. At the same time, the samples collected at the mountain site were transplanted to a different lowland habitat. Therefore, the lowland samples can be considered as control samples for the mountain ones. This experimental arrangement allows comparisons to be made between the treated and control samples and also between the mountain and lowland samples in each pair of treatments at each time period. Values at time zero were measured in thalli before treatment. Samples were recollected every half a year in spring and autumn to take seasonal advantage of the favourable light and humidity conditions for active metabolism (e.g. Lange 2003; Verseghy 1976). In addition, the high and low temperatures did not limit the photosynthetic activity in these seasons. Thalli of every treatment (treated and control samples: control lowland, treated lowland, control mountain, treated mountain) were recollected every half a year and dried in the laboratory. Photographs were taken regularly for documentation in the field.

Data of the Hungarian Meteorological Services, 2022 measured at stations near the experimental area (6.5 km from Tece and 7.5 km from Sóly) were analysed.

### Analysis of LSMs

Collected samples were checked for the presence of usnic acid and fumarprotocetraric acid by high-performance thin layer chromatography (HPTLC) according to standard methods for analysing lichen samples described by Arup et al. (1993) and Molnár and Farkas (2011). CAMAG horizontal chamber of 10 cm × 10 cm (DONAU LAB Kft., Budapest, Hungary), CAMAG TLC Plate Heater III (DONAU LAB Kft., Budapest, Hungary), and 10 cm × 10 cm thin-layer chromatographic plates (Merck, Kieselgel 60 F254) were used. Solvent system C (toluene: acetic acid, 20 : 3) was applied.

The amount of usnic acid and fumarprotocetraric acid was measured by high-performance liquid chromatography (HPLC, Alliance e2695, Waters Corporation, Milford, MA, USA) system, including a photodiode array detector (2998, Waters Corporation, Milford, MA, USA). Fifteen thalli were chosen from the randomised material of each treatment type in every sampling period. Thalli were c. 4–5 cm in diameter. The homogenised material (15 mg) was dissolved in 10 ml pure acetone and placed into an ultrasonic water bath for 10 min. The samples were then centrifuged for 20 min, and the supernatant was filtered through a Cronus Ø 25 mm PTFE syringe filter (0.22 μm). Standard stock solutions (1 mg ml^−1^) were made from reference standards for usnic acid (Sigma Aldrich Kft., Budapest, Hungary) and for fumarprotocetraric acid (Phytolab GmbH & Co. KG, Vestenbergsgreuth, Germany) dissolved in acetone for calibration purposes. The lichen metabolites were quantified according to a five-point (5, 10, 20, 50, 100 μg ml^−1^) calibration. The chromatographic method based on Ji and Khan (2005) was used. For chromatographic separation, a Phenomenex Luna 5 μm C18, 150 × 4.6 mm column was used, and 10 μl sample volume was injected. There was 40 °C in the column oven and 5 °C in the sample cooler. For the baseline separation of LSMs, a gradient elution program was used. Solvent A consisted of ortho-phosphoric acid and deionised (Milli-Q ultrapure) water (0.5 : 99.5), and solvent B contained ortho-phosphoric acid and acetonitrile (0.5 : 99.5). All the chemicals used were HPLC grade. The linear gradient started with a 60% A solvent after the volume decreased to 10% within 20 min and then to 0.5% in 30 s after which the volume remained constant for 9.5 min. The volume of solvent A was changed back to 60% within 1 min. The flow rate of solvents was 1 ml min^−1^. Lichen metabolites were detected at 280 nm (usnic acid) and 240 nm (fumarprotocetraric acid).

### Measurement of photosynthetic activity/vitality

Further thalli (*n* = 15) were chosen randomly from the recollected material. After cleaning, thalli were rehydrated by spraying with distilled water. Thalli were kept under low light (c. 10 μmol m^−2^ s^−1^) at seasonal ambient temperature for 1–2 days until the photosynthetic system regenerated (i.e. until *Fv*/*Fm* became constant). After 30 min of dark adaptation, chlorophyll *a* fluorescence kinetics were measured (described by Jensen 2002) on fully water-saturated lichen thalli with a portable pulse amplitude modulated fluorometer (FMS 2 Hansatech Instruments Ltd. UK; Modfluor software) in the laboratory. After 30 min of dark adaptation, the minimum fluorescence yield (*Fo*) was measured using a weak measuring beam for 3 s. The maximum fluorescence yield of the dark-adapted sample (*Fm*) was obtained with a saturation pulse of 7,500 μmol m^−2^ s^−1^ light intensity for 800 ms. From the measured parameters, maximum variable chlorophyll fluorescence yield in dark-adapted state (*Fv* = *Fm* − *Fo*) and maximum quantum yield of PSII photochemistry (*Fv*/*Fm* = (*Fm* − *Fo*) / *Fm*; Kitajima and Butler 1975) were calculated. After two additional saturating pulses were added for 800 ms, the maximum (*Fm*’) and the steady-state (*Ft*) fluorescence yields were determined. The Stern-Volmer non-photochemical quenching (NPQ), the yield of photochemical electron transport (φPSII), non-photochemical quenching (φNPQ), and the yield of non-regulated excitation dissipation (φNO) were calculated (Bilger and Björkman 1990; Kitajima and Butler 1975; Klughammer and Schreiber 2008). The last *Ft* and *Fm*’ values were used for calculations.

*Fv*/*Fm* and NPQ are the most frequently used chlorophyll fluorescence variables in ecological investigations. The values of *Fv*/*Fm* show the condition of the photosynthetic systems within the thalli and the efficiency of the photochemical reaction. The photobiont algae are self-protected from the harmful effects of high light intensity which cannot be used for photosynthesis (Demmig-Adams et al. 1989). The non-photochemical quenching represents the degree of these protective mechanisms (photoprotection) while photosynthesis is maintained. The values of φPSII give an insight into the effective photochemical quantum yield of PSII and describe the proportion of excitation energy used for charge separation. The φNPQ parameter represents the quantum yield of light-induced (ΔpH- and zeaxanthin-dependent) non-photochemical fluorescence quenching. The φNO parameter shows the combined pathways of radiative and non-radiative deexcitation reactions that do not lead to photochemical energy conversion and are not involved in the NPQ mechanisms (Klughammer and Schreiber 2008). The sum of these complementary processes equals 1 (Kramer et al. 2004).

Usually, the chlorophyll fluorescence and HPLC measurements were started a few days after collection, except in the case of samples collected in spring 2020 because of the COVID-19 situation. The investigations had to be delayed, and thalli deteriorated during that time.

### Statistical analysis

**Fig. 2:**
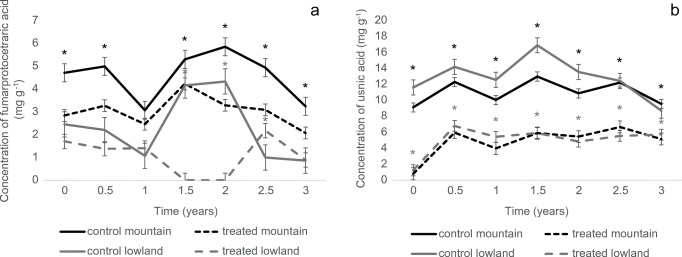
**a**–**b** The concentration of fumarprotocetraric acid (**a**) and usnic acid (**b**) in the acetone rinsed and control samples of *Cladonia foliacea* deriving from the lowland and mountain habitats during a 3-year field experiment. Significant differences between the treated and control thalli are marked with asterisks. Sample size: mountain treated = 105, mountain control = 105, lowland treated = 105, lowland control = 105. The timepoint zero refers to the state when every thallus was still before treatment

**Fig. 3:**
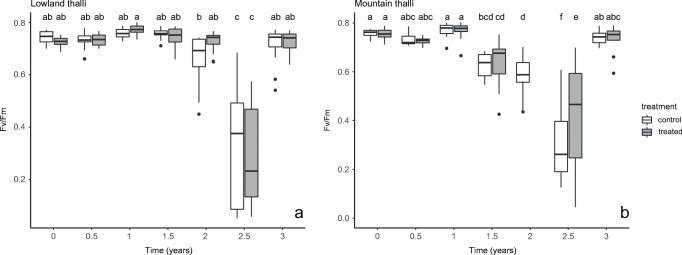
**a**–**b** The photosynthetic activity (*Fv*/*Fm*) of the acetone rinsed (= treated) and control samples of *Cladonia foliacea* deriving from the lowland (**a**) and mountain (**b**) habitats during a 3-year field experiment. Treatments with the same letter are not significantly different at 95% confidence. Sample size: mountain treated = 88, mountain control = 96, lowland treated = 105, lowland control = 97. The timepoint zero refers to the state when every thallus was still before treatment

**Fig. 4:**
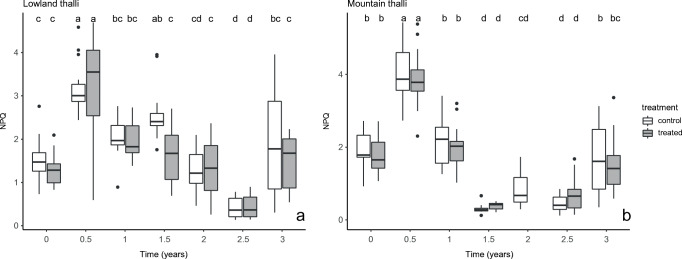
**a**–**b** The non-photochemical quenching (NPQ) values of the acetone rinsed (=treated) and control samples of *Cladonia foliacea* deriving from the lowland (**a**) and mountain (**b**) habitats during a 3-year field experiment. Treatments with the same letter are not significantly different at 95% confidence. Sample size: mountain treated = 88, mountain control = 96, lowland treated = 105, lowland control = 97. The timepoint zero refers to the state when every thallus was still before treatment

The effect of seasons (autumn and spring), locality (lowland, mountain), and treatment (acetone rinsed, control) on usnic acid, fumarprotocetraric acid, and the values of *Fv*/*Fm* and NPQ were statistically evaluated. The statistical analyses were carried out with the R software version 3.6.3 (R Core Team 2020). The effect of long-term (site) and short-term (season) environmental changes and treatments on LSMs concentration was tested by a two-way ANOVA followed by a Tukey HSD test. The normality of data distribution was checked visually with a Q-Q plot (quantiles of the residuals are plotted against the quantiles of the normal distribution with a 45° degree reference line) and by Shapiro-Wilk normality test. The homogeneity of variances was tested by Levene’s test. A level of *p* < 0.05 was considered a significant difference. Graphs were prepared in MS Excel (Fig. 2) and R environment (Figs. 3, 4 and Supplementary Figure S1).

## Results

### The concentrations of usnic acid and fumarprotocetraric acid

#### Difference between the lowland and mountain thalli

During the whole investigation period, there was a highly significant difference in fumarprotocetraric acid and usnic acid concentrations between the lowland and mountain thalli, both in the control and treated samples (Supplementary Table S1). The fumarprotocetraric acid concentration was higher in the mountain than in the lowland samples, whereas the usnic acid concentration was significantly higher than in the mountain thalli (Fig. 2).

#### Difference between the treated and control samples

The control samples showed significantly higher fumarprotocetraric acid (*p* < 0.001 for mountain and lowland, respectively) (Fig. 2a) and usnic acid concentrations (*p* < 0.001 for mountain and lowland, respectively, Fig. 2b) than the treated samples deriving from both sites (Supplementary Table S2). The ranges of fumarprotocetraric acid concentrations were 2.07–4.22 mg g^−1^ in the treated mountain thalli (*n* = 105) and 1.08–2.17 mg g^−1^ in the treated lowland (*n* = 105) thalli; the control samples showed values of 3.06–5.85 mg g^−1^ in the mountain thalli (*n* = 105) and 0.86–4.33 mg g^−1^ in the lowland samples (*n* = 105). Usnic acid concentrations were 0.81–6.66 mg g^−1^ in the treated mountain thalli (*n* = 105) and 1.24–6.79 mg g^−1^ in the treated lowland (*n* = 105) thalli; control values were 9.1–12.97 mg g^−1^ in the mountain thalli and 8.7–16.89 mg g^−1^ in the lowland thalli. After half a year, the concentration of usnic acid achieved a saturation value when its amount did not rise significantly in the treated samples during the following years (Fig. 2b), being only half of the values measured in the control thalli. The trends in the production of fumarprotocetraric acid are not so clear: the treated lowland samples from 1.5 to 2 years did not show the seasonal trends characterising the other samples (Fig. 2a).

#### The effect of seasonality on LSM concentration

There was a seasonal fluctuation of the LSMs in the control and treated samples (Fig. 2). The fumarprotocetraric acid concentrations were significantly higher (*p* < 0.0001) in spring than in autumn in the mountain, unlike in the lowland (*p* = 0.415) samples (Supplementary Table S2). A significantly higher level of usnic acid was measured in spring than in autumn collected samples (*p* < 0.0001 for the lowland and mountain thalli, Supplementary Table S2).

### Chlorophyll fluorescence parameters

The lowland and mountain control thalli did not differ significantly (*p* = 1.00, Supplementary Table S1) in *Fv*/*Fm* at the start of the experiment. Control and treated samples did not show significant differences in *Fv*/*Fm* in the lowland (*p* = 0.675, *n*_control_ = 97, *n*_treated_ = 105) or mountain (*p* = 0.665, *n*_control_ = 96, *n*_treated_ = 88) thalli (Fig. 3, Supplementary Table S3). The values showed a seasonal fluctuation in the mountain (*p* < 0.001) and lowland (*p* < 0.001) samples (Fig. 3, Supplementary Table S3). The *Fv*/*Fm* was higher in autumn than in spring samples (Fig. 3). There were no significant differences in *Fv*/*Fm* between the lowland and mountain samples, except for the control thalli of the second spring (*p* = 0.05) (Supplementary Table S1).

There was no significant difference in NPQ between the lowland and mountain control thalli at the start of the experiment (*p* = 0.99, Supplementary Table S1). The NPQ did not differ significantly between the control and treated thalli during the investigation period (mountain: *p* = 0.769; lowland: *p* = 0.064) (Fig. 4, Supplementary Table S3). The seasons affected the level of NPQ in the lowland samples (*p* = 0.0027), unlike those in the mountain (*p* = 0.422) samples (Fig. 4, Supplementary Table S3). The spring samples showed higher values than the autumn samples (Fig. 4). Lowland and mountain samples did not differ significantly in NPQ, except for the thalli of the second spring (*p* < 0.0001) (Supplementary Table S1).

There was no significant difference in φPSII between the lowland and mountain control thalli at the start of the experiment (*p* = 0.098, Supplementary Table S1). Control and treated samples did not show significant differences in φPSII in the lowland (*p* = 0.64) or mountain (*p* = 0.97) thalli (Supplementary Table S3). The values differed significantly between autumn and spring in the mountain (*p* = 0.003) and lowland (*p* = 0.04) samples (Supplementary Table S3). There were no significant differences in φPSII between the lowland and mountain samples during the investigation period, except for the thalli of the second spring (*p* < 0.0001) (Supplementary Table S1).

There was no significant difference in φNPQ between the lowland and mountain control thalli at the start of the experiment (*p* = 0.45, Supplementary Table S1). Control and treated samples did not show significant differences in φNPQ in the lowland (*p* = 0.16) or mountain (*p* = 0.85) thalli (Supplementary Table S3). The values differed significantly between autumn and spring in the mountain samples (*p* = 0.0003, autumn > spring) and lowland samples (*p* = 0.025, autumn < spring) (Supplementary Table S3). The φNPQ was usually higher in autumn than in spring collected thalli (Table 1). There were no significant differences in φNPQ between the lowland and mountain samples, except for the thalli of the second spring (*p* < 0.0001 for control and *p* = 0.04 for treated thalli) (Supplementary Table S1).

**Table 1:** Mean partition (%) of incoming light energy between photochemical quenching (φPSII), regulated non-photochemical quenching (φNPQ), and non-regulated excitation dissipation (φNO) in the acetone-treated and control samples of *Cladonia foliacea*

Time	Treated lowland			Control lowland			Treated mountain			Control mountain		
(Years)	φPSII	φNPQ	φNO	φPSII	φNPQ	φNO	φPSII	φNPQ	φNO	φPSII	φNPQ	φNO
0	20% ± 5%*	44% ± 5%	36% ± 6%	31% ± 4%	41% ± 6%	28% ± 5%	22% ± 7%	49% ± 6%	29% ± 6%	22% ± 5%	51% ± 7%	27% ± 5%
0.5	30% ± 6%	53% ± 8%	18% ± 5%	29% ± 5%	54% ± 5%	17% ± 3%	27% ± 4%	58% ± 5%	15% ± 3%	27% ± 3%	59% ± 4%	15% ± 2%
1	29% ± 8%	44% ± 5%	27% ± 8%	30% ± 6%	45% ± 6%	25% ± 7%	24% ± 10%	50% ± 10%	26% ± 6%	29% ± 9%	48% ± 8%	24% ± 6%
1.5	27% ± 13%	38% ± 10%*	34% ± 17%	22% ± 16%	42% ± 12%	36% ± 20%	12% ± 5%	24% ± 5%	64% ± 5%	4% ± 9%	25% ± 11%	71% ± 8%
2	11% ± 9%	32% ± 14%	57% ± 21%	16% ± 8%	43% ± 10%	41% ± 14%				14% ± 5%	37% ± 12%	49% ± 12%
2.5	6% ± 6%	23% ± 8%	71% ± 13%	4% ± 7%	10% ± 13%	27% ± 35%	13% ± 7%	31% ± 11%	55% ± 17%	6% ± 8%	18% ± 16%	42% ± 33%
3	18% ± 9%	48% ± 9%	35% ± 13%	17% ± 8%	47% ± 11%	36% ± 16%	17% ± 9%	47% ± 7%	36% ± 12%	19% ± 7%	45% ± 12%	36% ± 17%

Thalli were derived from the lowland and mountain habitats and, after the treatment, kept under the same field conditions for 3 years (sample size: mountain treated = 88, mountain control = 96, lowland treated = 105, lowland control = 97). Significant differences between the control and treated samples (within lowland or mountain thalli) are depicted with asterisks. The timepoint zero referred to the situation when every thallus was still before treatment

There was no significant difference in φNO between the lowland and mountain control thalli at the start of the experiment (*p* = 1.00, Supplementary Table S1). Control and treated samples did not show significant differences in φNO in the lowland (*p* = 0.22) or mountain (*p* = 0.88) thalli (Supplementary Table S3). The values differed significantly between autumn and spring in the mountain samples (*p* = 0.0001), unlike those in the lowland (*p* = 0.89) samples. There were no significant differences in φNO between the lowland and mountain samples, except for the thalli of the second spring (*p* < 0.0001) (Supplementary Table S1).

### The appearance of lichenicolous fungi

**Fig. 5:**
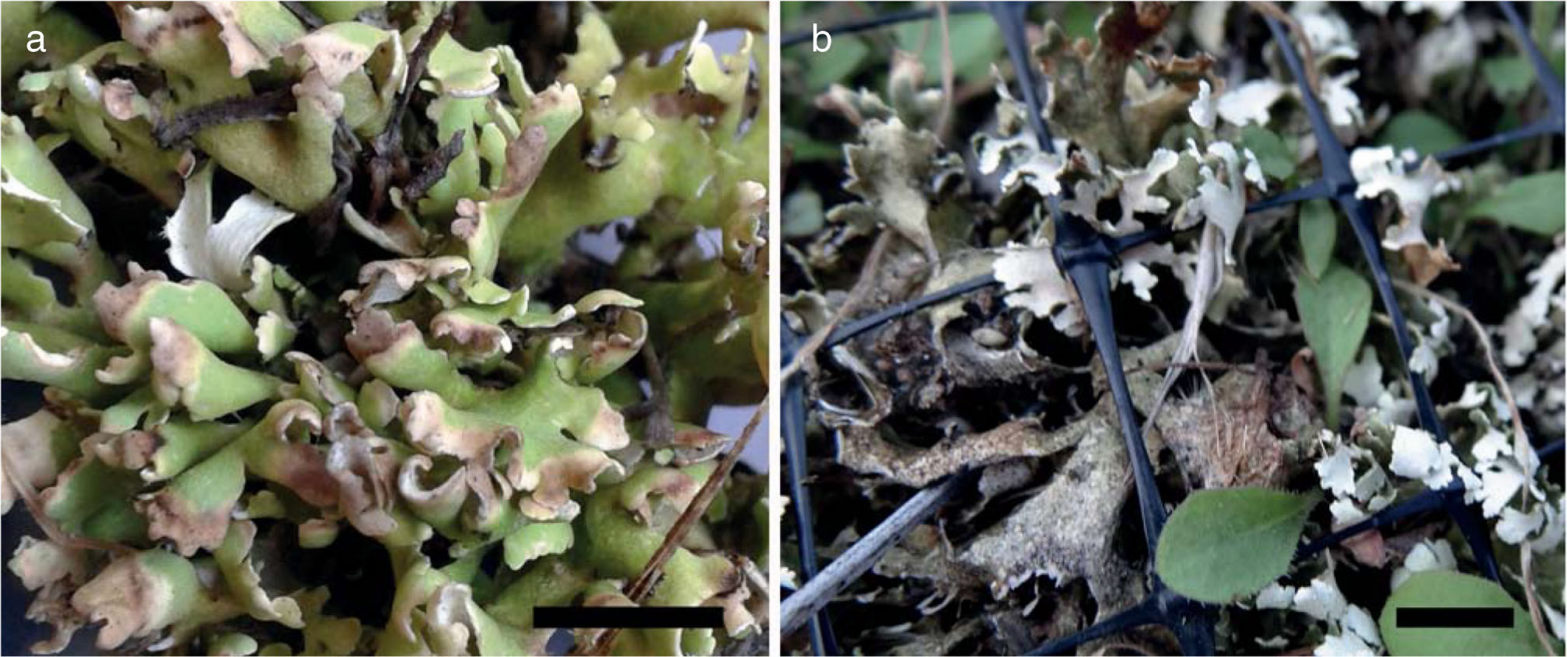
**a**–**b** Lichenicolous fungi on thalli of *Cladonia foliacea* observed as necrotic patches at lobe ends (**a**) or as continuous spots (**b**) the line represents a 1 cm scale

After half a year of transplantation, black necrotic lines and necrotic patches of various sizes were observed on several acetone rinsed transplanted thalli originating from the lowland site (Fig. 5), either at lobe ends (c. 0.5 cm^2^) (Fig. 5a) or later as larger continuous spots (Fig. 5b) laminally, due to infection by lichenicolous fungi. They grew to colonise entire thalli over time. Investigating the non-transplanted control samples, it was found that the lichens were already infected by the fungi upon collection, although it was less obvious due to the small size of the necrotic patches (less than c. 0.5 cm^2^) and the low abundance (most of the thalli did not appear to be infected). The lichenicolous fungus found on both the control and acetone-treated thalli originating either from lowland or mountain habitats was identified as *Didymocyrtis cladoniicola* (Diederich, Kocourk. & Etayo) Ertz & Diederich (Diederich et al. 2007, Ertz et al. 2015). The lichenicolous *Syspastospora cladoniae* Etayo (Etayo 2008) was only found on one of the lowland control thalli and not observed on the transplanted samples.

## Discussion

### The long-term effect of acetone rinsing

Our results highlight that lower levels of usnic acid are present in the acetone treated than in the control thalli even after 3 years under field conditions in the lowland and mountain samples. The results suggested that the levels of usnic acid did not rise further after half a year, only fluctuating around a saturation value between the seasons. However, the difference in levels between the control and treated samples showed a slight decrease. After 3 years, the control samples contained about two times more usnic acid than the treated thalli indicating that usnic acid production needs a more extended period for regeneration after acetone treatment. At the same time, the amount of usnic acid proved to be enough to protect the thalli from damage to the photosynthetic system, as noted, for example, by Solhaug et al. (2003).

Our results also revealed that the control and treated samples did not show significant differences in *Fv*/*Fm*, NPQ, φPSII, φNPQ, and φNO, indicating a similarly good condition of all the samples. The photosynthetic systems worked on a high level and could safely dissipate the excessive light energy mainly in a regulated way (φNPQ > φNO), except in the first and second spring, which were extremely dry seasons (Supplementary Figure S1). Therefore, the thalli of *Cladonia foliacea* can survive for years after acetone treatment. Veres et al. (2022) pointed out that *C. foliacea* contained approximately four times more cortical pigment (usnic acid) compared to, for example, *C. furcata* or *C. magyarica* containing atranorin in their cortex derived from the same microhabitat collected at the same time. Probably the usnic acid can significantly contribute to the survival of *C. foliacea* in habitats with extreme irradiation and drought, helping the thalli against photodamage. Furthermore, the species can avoid photoinhibition by curling during desiccation (Barták et al. 2006), providing an advantage over other species. In the desiccated state, the white medulla and the fumarprotocetraric acid crystals on the hyphae can reflect radiation, thereby protecting algae against photodamage.

The lichenicolous fungus *Didymocyrtis cladoniicola* was relatively more abundant on acetone rinsed than on control thalli; therefore, it is concluded that the acetone treatment could have an effect on the relation of the lichens and the lichenicolous fungus, as also mentioned by Bergmann and Werth (2017). Since the fungus could spread more easily on thalli with lowered levels of usnic acid in the cortex and fumarprotocetraric acid in the medulla due to acetone treatment, the antifungal role of usnic acid and fumarprotocetraric acid can be hypothesised. It corresponds well with former results on influencing lichen fitness by lichenicolous fungi via reducing their growth rates (Asplund et al. 2018, Lawrey 1993, Merinero and Gauslaa 2018). Lawrey et al. (1994) and Lawrey (2000) explain easier colonisation on the lichen thallus by enzymatic degradation of lichen secondary metabolites from the lichenicolous fungi.

### The effect of transplantation

Usnic acid showed higher concentrations in the lowland than in the mountain samples during the whole investigation period. The lowland samples were derived from more open vegetation than the mountain samples, where less irradiation could reach lichen thalli, proved by the lower level of usnic acid at the start of the experiment in the control samples. Probably the level of photoprotection could be better explained by a long-term adaptation than a short-term acclimation. A higher amount of UV-protectant lichen metabolite indicates a higher level of incoming irradiation in thalli growing under humid conditions (Nybakken and Julkunen-Tiitto, 2006; Solhaug et al. 2003) and thus an elevated level of need in photoprotection (Heber et al. 2006). The LSMs can be produced only by rehydrated thalli (Solhaug et al. 2003) and need enough photosynthates for production (Solhaug and Gauslaa 2004). There were no significant differences in *Fv*/*Fm* and φPSII between the lowland and mountain control thalli at the start and during the experiment, indicating that the photosynthetic activity did not differ and the amount of photosynthates needed for LSM production could also reach the same level. Since the thalli of various treatments were only within a c. 0.5 m distance in the experimental area, the very similar microenvironmental conditions could not cause a significant difference in the function of PSII. The difference in the LSM production between the lowland and mountain thalli can be explained by a long-term adaptation of the fungi (on a genetic, anatomical, or structural level) that did not change with transplantation. NPQ and φNPQ also showed similar levels, suggesting that the amount of light reaching algal cells must be the same, triggering the same level of response in the algal photoprotection. Therefore, a thicker cortical layer was probably exhibited in the lowland samples containing more cortical pigment (but needs further investigation). We also detected the sudden increase of significant differences between the lowland and mountain samples (collected/measured) after 1.5 years. We did not find any differences in the soil microenvironment, shade conditions, or the conditions of the thalli compared to other thalli collected earlier or remaining on the field.

The lichenicolous fungus *Didymocyrtis cladoniicola* found both in the control and treated thalli originating from the lowland and mountain habitats was more and more obvious over time. It was hardly visible at the time of transplantation and reached a detectable size after 6 months in the lowland and 12 months in the mountain thalli. However, the transplantation probably caused a slight change that was advantageous for the growth of the fungus, and thus, their appearance became more obvious (reaching more than 50 cm^2^ area of the thalli — at various treatments) during the experimental period. Therefore, the increased growth of lichenicolous fungi is a possible effect of the disturbance caused by the transplantation itself.

### The effect of seasonality on the production of LSMs

There was a seasonal fluctuation of the LSMs in the control and treated mountain and lowland lichens. A significantly higher level of usnic acid was measured in spring than in autumn collected samples. The seasonal change in usnic acid concentration was compared with meteorological data (the means of 3 months before sampling) (Supplementary Figure S1). It was revealed that the usnic acid level, especially in mountain samples, mainly reflected the changes in relative humidity (spring > autumn), unlike global irradiation (spring < autumn). Moistened *Xanthoria parietina* re-synthesised 15–25% of their parietin content within 3 weeks after acetone rinsing between field conditions, and thalli kept under dry conditions did not produce parietin regardless of the amount of incoming irradiation (Solhaug et al. 2003). Our results also showed that the amount of available humidity largely affected the production of cortical usnic acid concentration in the investigated semi-arid region on a long-term scale. Between more balanced humidity conditions, the available global irradiation possibly limits the production of cortical UV protective pigments (e.g. Nybakken and Julkunen-Tiitto 2006). Probably the thinner cortical layer of the mountain thalli could cause them to reflect more sensitively the seasonal changes of the environment (Veres et al. 2022), but this needs further evidence.

The seasons also affected the values of *Fv*/*Fm*, NPQ, and the proportion of incoming light energy between the quenching mechanisms. In spring, lower *Fv*/*Fm* and higher NPQ and φNO were measured than in the thalli collected in autumn. These phenomena indicated a higher need for photoprotection and a lowered photosynthetic activity during spring, probably caused by a higher level of incoming irradiation reaching algal cells. The higher relative humidity during spring (Supplementary Figure S1) may result in the longer time spent in unfolded state and an elevated cortex transmittance (Dietz et al. 2000). The φNO values were higher in spring than in autumn, suggesting that the excessive light energy was dissipated mainly in a non-regulated way (φNO > φNPQ). In semi-arid grasslands, a lichen shows its highest photosynthetic activity (Farkas et al. 2020, Veres et al. 2020) and biomass production (Verseghy 1976) during autumn in Hungary, in agreement with our current results. It was assumed that the higher photosynthetic activity and biomass production might allow an elevated level of photosynthates used for LSM production. However, Gauslaa et al. (2013) demonstrated that growth and defence by LSMs (e.g. against herbivory) are such investments that did not compete for photosynthates.

It can be concluded that the algae were more sensitive to the seasonally changing environment than the acetone treatment. The treatment mostly affected the mycobiont partner and the algae did not elevate their photoprotective role (φNO and φNPQ did not differ in the control and treated thalli), as Veres et al. (2022) pointed out in *Cladonia furcata*. Probably the initial amount of UV-protectant lichen metabolite originated from a high level of constitutive photoprotection (Asplund et al. 2009), and the system was over-secured. After the treatment, a lower amount of usnic acid would be enough for the necessary level of UV defence. In every measured chlorophyll fluorescence parameter, the seasonal fluctuation was more pronounced in the mountain than in the lowland samples. The mountain thalli reflected the environmental changes more intensely than the lowland samples, probably because of their sensitivity deriving from a thinner cortex (needs further evidence) impacting water holding capacity (Verseghy 1971).

The monitoring of the usnic acid production within a half year would reveal further details on/about the intensity of reproduction of this metabolite under semi-arid field conditions and from the climate change point of view. The scenario that the severity and frequency of drought events will be increasing in some regions (Bartholy et al. 2009; Ferrenberg et al. 2015) raises the question of how terricolous lichen will or could acclimate and adapt to the changing environment. The reduced rehydration could significantly decrease the vitality of terricolous lichens (Morillas et al. 2021) and thereby create less chance for UV-protectant metabolite production. The investigation of the long-term effect of acetone rinsing on other species containing various UV-protectant metabolites or living in different habitats would expand our knowledge on this topic.

## Supplementary information

ESM 1 (DOCX 429 kb)

## Data Availability

The datasets analysed during the current study are available from the corresponding author on reasonable request. Lichen specimens are deposited in the Lichen Herbarium VBI (Vácrátót, Hungary).
