# Supplementary material for: The long-term effect of removing the UV-protectant usnic acid from the thalli of the lichen Cladonia foliacea
Source: Mycol Prog. 2022 Sep 1;21(9):83. doi: 10.1007/s11557-022-01831-y (PMC9433529; doi:10.1007/s11557-022-01831-y)
Supplement: Supplementary file 1 — (DOCX 429 kb) [file 11557_2022_1831_MOESM1_ESM.docx]

Supplementary material

The long term effect of removing the UV protectant usnic acid from the thalli of the lichen *Cladonia foliacea*

Katalin Veres, Mónika Sinigla, Krisztina Szabó, Nóra Varga, Edit Farkas

**Supplementary Table S1** The results of ANOVA showing significant differences between mountain and lowland samples in fumarprotocetraric acid, usnic acid, photosynthetic activity (Fv/Fm), non-photochemical quenching (NPQ), the proportion of incoming light energy between photochemical quenching (φPSII), regulated non-photochemical quenching (φNPQ) and non-regulated energy dissipation (φNO) parameters in treated (t) and control (c) *Cladonia foliacea* thalli during the three-year investigation period. Sample size of the different measurements were as follows: mountain treated = 88, mountain control = 96, lowland treated = 105, lowland control = 97 for chlorophyll fluorescence measurements and mountain treated = 105, mountain control = 105, lowland treated = 105, lowland control = 105 for LSM concentration measurements during the investigation period.

| **years** | **0** | **0.5** | **1** | **1.5** | **2** | **2.5** | **3** |
| --- | --- | --- | --- | --- | --- | --- | --- |
| fumarprotocetraric acid (t) | p<0.0001 | p<0.0001 | p<0.0001 | p<0.0001 | p<0.0001 | p<0.0001 | p<0.0001 |
| fumarprotocetraric acid (c) | p<0.0001 | p<0.0001 | p<0.0001 | 0.0097 | 0.0001 | p<0.0001 | p<0.0001 |
| usnic acid (t) | 0.39 | 0.0006 | p<0.0001 | p<0.0001 | 0.026 | p<0.0001 | 0.074 |
| usnic acid (c) | p<0.0001 | p<0.0001 | p<0.0001 | p<0.0001 | p<0.0001 | 0.99 | 0.298 |
| Fv/Fm (t) | 0.99 | 1.00 | 1.00 | 0.07 | na | 0.002 | 1.00 |
| Fv/Fm (c) | 1.00 | 1.00 | 1.00 | 0.05 | 0.84 | 1.00 | 1.00 |
| NPQ (t) | 0.91 | 0.64 | 1.00 | p<0.0001 | na | 0.99 | 1.00 |
| NPQ (c) | 0.99 | 0.02 | 1.00 | p<0.0001 | 0.99 | 1.00 | 0.99 |
| φPSII (t) | 0.99 | 0.95 | 0.04 | p<0.0001 | na | 0.81 | 1.00 |
| φPSII (c) | 0.098 | 0.59 | 0.98 | p<0.0001 | 1.00 | 1.00 | 0.99 |
| φNPQ (t) | 0.99 | 0.91 | 0.98 | 0.04 | na | 0.99 | 1.00 |
| φNPQ (c) | 0.45 | 0.73 | 0.99 | p<0.0001 | 0.79 | 1.00 | 1.00 |
| φNO (t) | 0.96 | 1.00 | 1.00 | p<0.0001 | na | 0.28 | 1.00 |
| φNO (c) | 1.00 | 1.00 | 1.00 | p<0.0001 | 0.89 | 1.00 | 1.00 |

na = no data available

Supplementary Table S2. The output of the two-way ANOVA analysis showing the combined effect of treatment and seasons on lowland and mountain thalli of *Cladonia foliacea* on fumarprotocetraric acid and usnic acid concentration. df = degrees of freedom, sum sq = sum of squares, mean sq = mean of the sum squares, signif. level = Significance codes: 0 ‘***’ 0.001 ‘**’ 0.01 ‘*’ 0.05 ‘.’ 0.1 ‘ ’ 1 Sample size of the different measurements were as follows: mountain treated = 88, mountain control = 96, lowland treated = 105, lowland control = 97 for chlorophyll fluorescence measurements and mountain treated = 105, mountain control = 105, lowland treated = 105, lowland control = 105 for LSM concentration measurements during the investigation period.

|  | **Df** | **Sum Sq** | **Mean Sq** | **F value** | **Pr(>F)** | **signif. level** |
| --- | --- | --- | --- | --- | --- | --- |
| fumarprotocetraric acid - mountain |  |  |  |  |  |  |
| treatment | 1 | 42.17 | 42.17 | 63.25 | 3.21e-11 | *** |
| season | 1 | 12.65 | 12.65 | 18.98 | 4.73e-05 | *** |
| treatment : season | 1 | 0.00 | 0.00 | 0.00 | 0.99 |  |
|  |  |  |  |  |  |  |
| lowland |  |  |  |  |  |  |
| treatment | 1 | 25.83 | 25.827 | 19.410 | 3.96e-05 | *** |
| season | 1 | 0.90 | 0.897 | 0.674 | 0.415 |  |
| treatment : season | 1 | 0.03 | 0.035 | 0.026 | 0.872 |  |
|  |  |  |  |  |  |  |
| usnic acid - mountain |  |  |  |  |  |  |
| treatment | 1 | 665.6 | 665.6 | 494.807 | <2.00e-16 | *** |
| season | 1 | 104.2 | 104.2 | 77.457 | 9.82e-13 | *** |
| treatment : season | 1 | 0.4 | 0.4 | 0.261 | 0.611 |  |
|  |  |  |  |  |  |  |
| lowland |  |  |  |  |  |  |
| treatment | 1 | 1059.6 | 1059.6 | 360.54 | <2.00e-16 | *** |
| season | 1 | 94.0 | 94.0 | 31.98 | 3.61e-07 | *** |
| treatment : season | 1 | 5.4 | 5.4 | 1.84 | 0.18 |  |

Supplementary Table S3. The output of the two-way ANOVA analysis showing the combined effect of treatment and seasons on lowland and mountain thalli of *Cladonia foliacea* on Fv/Fm, NPQ, φPSII, φNPQ and φNO. df = degrees of freedom, sum sq = sum of squares, mean sq = mean of the sum squares, signif. level = Significance codes: 0 ‘***’ 0.001 ‘**’ 0.01 ‘*’ 0.05 ‘.’ 0.1 ‘ ’ 1 Sample size of the different measurements were as follows: mountain treated = 88, mountain control = 96, lowland treated = 105, lowland control = 97 for chlorophyll fluorescence measurements and mountain treated = 105, mountain control = 105, lowland treated = 105, lowland control = 105 for LSM concentration measurements during the investigation period.

|  | **Df** | **Sum Sq** | **Mean Sq** | **F value** | **Pr(>F)** | **signif. level** |
| --- | --- | --- | --- | --- | --- | --- |
| Fv/Fm - mountain |  |  |  |  |  |  |
| treatment | 1 | 0.003 | 0.0034 | 0.188 | 0.665 |  |
| season | 1 | 0.883 | 0.8832 | 48.151 | 6.7e-11 | *** |
| treatment : season | 1 | 0.016 | 0.0162 | 0.883 | 0.349 |  |
|  |  |  |  |  |  |  |
| lowland |  |  |  |  |  |  |
| treatment | 1 | 0.004 | 0.0041 | 0.176 | 0.675 |  |
| season | 1 | 0.611 | 0.6109 | 26.218 | 7.14e-07 | *** |
| treatment : season | 1 | 0.036 | 0.0364 | 1.564 | 0.212 |  |
|  |  |  |  |  |  |  |
| NPQ - mountain |  |  |  |  |  |  |
| treatment | 1 | 0.2 | 0.1512 | 0.086 | 0.769 |  |
| season | 1 | 1.2 | 1.1358 | 0.649 | 0.422 |  |
| treatment : season | 1 | 1.8 | 1.7942 | 1.024 | 0.313 |  |
|  |  |  |  |  |  |  |
| lowland |  |  |  |  |  |  |
| treatment | 1 | 3.51 | 3.509 | 3.460 | 0.06436 | . |
| season | 1 | 9.38 | 9.379 | 9.245 | 0.00268 | ** |
| treatment : season | 1 | 0.45 | 0.454 | 0.447 | 0.50438 |  |
|  |  |  |  |  |  |  |
| φPSII - mountain |  |  |  |  |  |  |
| treatment | 1 | 0.0000 | 0.00001 | 0.001 | 0.97065 |  |
| season | 1 | 0.0736 | 0.07363 | 8.856 | 0.00332 | ** |
| treatment : season | 1 | 0.0012 | 0.00120 | 0.144 | 0.70459 |  |
|  |  |  |  |  |  |  |
| lowland |  |  |  |  |  |  |
| treatment | 1 | 0.0029 | 0.00294 | 0.220 | 0.6395 |  |
| season | 1 | 0.0567 | 0.05669 | 4.244 | 0.0407 | * |
| treatment : season | 1 | 0.0029 | 0.00292 | 0.218 | 0.6407 |  |
|  |  |  |  |  |  |  |
| φNPQ - mountain |  |  |  |  |  |  |
| treatment | 1 | 0.001 | 0.00064 | 0.035 | 0.852720 |  |
| season | 1 | 0.254 | 0.25427 | 13.658 | 0.000291 | *** |
| treatment : season | 1 | 0.026 | 0.02553 | 1.372 | 0.243079 |  |
|  |  |  |  |  |  |  |
| lowland |  |  |  |  |  |  |
| treatment | 1 | 0.0264 | 0.02637 | 2.014 | 0.1574 |  |
| season | 1 | 0.0670 | 0.06704 | 5.120 | 0.0247 | * |
| treatment : season | 1 | 0.0251 | 0.02506 | 1.914 | 0.1681 |  |
|  |  |  |  |  |  |  |
| φNO - mountain |  |  |  |  |  |  |
| treatment | 1 | 0.001 | 0.0008 | 0.022 | 0.883200 |  |
| season | 1 | 0.602 | 0.6016 | 15.805 | 0.000102 | *** |
| treatment : season | 1 | 0.016 | 0.0156 | 0.411 | 0.522482 |  |
|  |  |  |  |  |  |  |
| lowland |  |  |  |  |  |  |
| treatment | 1 | 0.047 | 0.04730 | 1.507 | 0.221 |  |
| season | 1 | 0.001 | 0.00062 | 0.020 | 0.888 |  |
| treatment : season | 1 | 0.011 | 0.01110 | 0.354 | 0.553 |  |


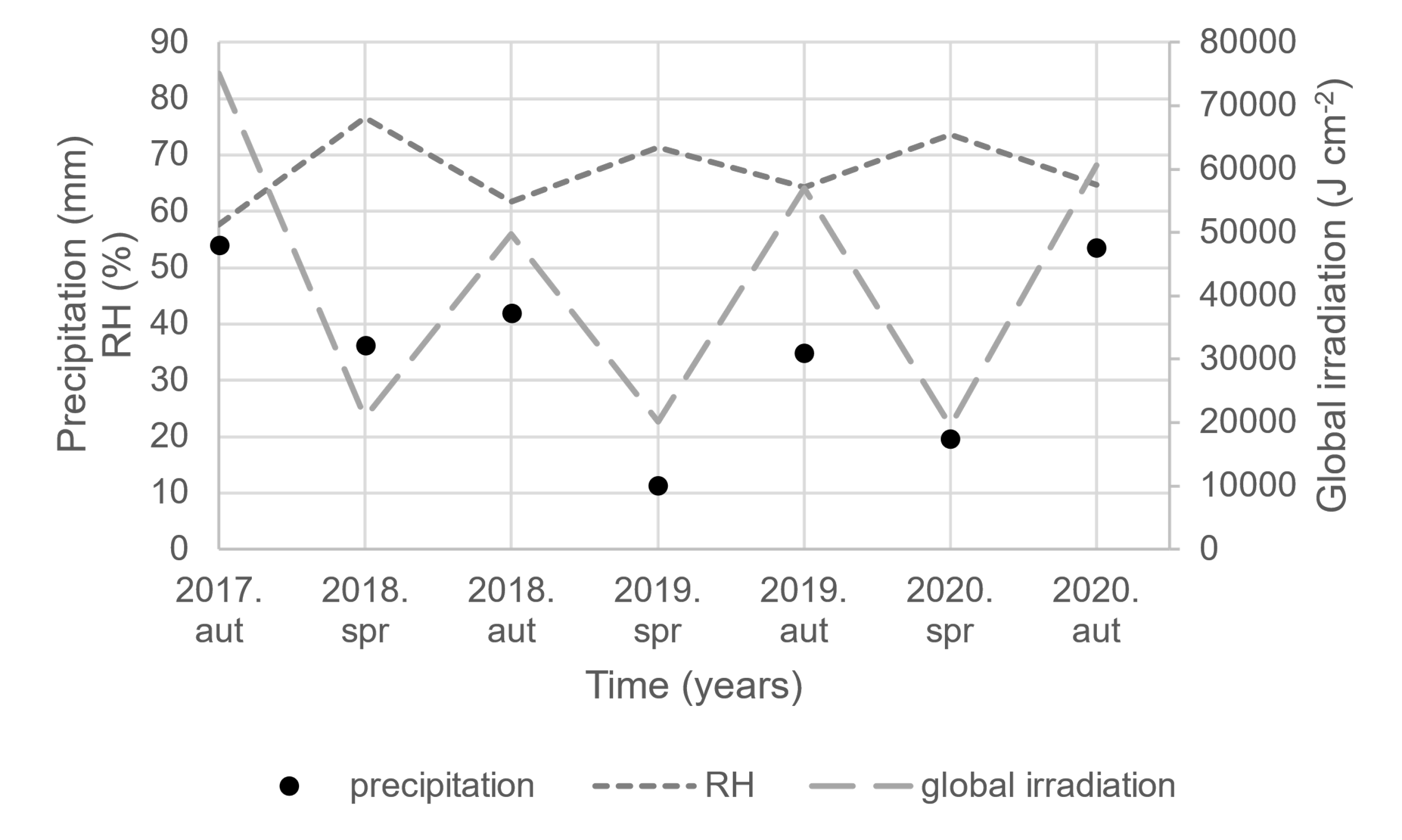


Supplementary Figure S1 Meteorological data during the three-year experiment: a three month average of sum global irradiation, precipitation and a three month average of relative humidity (RH) before sampling
